# Response Properties of the Auditory Telencephalon in Songbirds Change with Recent Experience and Season

**DOI:** 10.1371/journal.pone.0002854

**Published:** 2008-08-06

**Authors:** Thomas A. Terleph, Kai Lu, David S. Vicario

**Affiliations:** 1 Biology Department, Sacred Heart University, Fairfield, Conneticut, United States of America; 2 Psychology Department, Rutgers University, Piscataway, New Jersey, United States of America; Max-Planck-Institut fuer Neurobiologie, Germany

## Abstract

The caudomedial nidopallium (NCM) is a telencephalic auditory area that is selectively activated by conspecific vocalizations in zebra finches and canaries. We recently demonstrated that temporal and spectral dynamics of auditory tuning in NCM differ between these species [1]. In order to determine whether these differences reflect recent experience, we exposed separate groups of each species and sex to different housing conditions. Adult birds were housed either in an aviary with conspecifics (NORM), with heterospecifics (canary subjects in a zebra finch aviary, and vice versa: (CROSS)), or in isolation (ISO) for 9 days prior to testing. We then recorded extracellular multi-unit electrophysiological responses to simple pure tone stimuli (250–5000 Hz) in awake birds from each group and analyzed auditory tuning width using methods from our earlier studies. Relative to NORM birds, tuning was narrower in CROSS birds, and wider in ISO birds. The trend was greater in canaries, especially females. The date of recording was also included as a covariate in ANCOVAs that analyzed a larger set of the canary data, including data from birds tested outside of the breeding season, and treated housing condition and sex as independent variables. These tests show that tuning width was narrower early in the year and broader later. This effect was most pronounced in CROSS males. The degree of the short-term neural plasticity described here differs across sexes and species, and may reflect differences in NCM's anatomical and functional organization related to species differences in song characteristics, adult plasticity and/or social factors. More generally, NCM tuning is labile and may be modulated by recent experience to reflect the auditory processing required for behavioral adaptation to the current acoustic, social or seasonal context.

## Introduction

Songbirds learn their songs through a process of vocal imitation that has much in common with human speech acquisition [2]. Songbirds sing songs with both species-specific and individually-unique features, due to the learning process, and use these songs to communicate in social and reproductive contexts (reviews in [3]). Caudomedial nidopallium (NCM, formerly caudomedial neostriatum), is a telencephalic region of the songbird brain [4] that contributes to the processing of these communication signals, and is selectively activated by complex sounds [5]. In all avian species studied to date, including the two most commonly used in laboratory studies of birdsong neurobiology, the zebra finch (*Taeniopygia guttata*) and canary (*Serinus canaria*), the immediate early gene *zenk* is rapidly expressed in NCM in response to birdsong. ZENK induction in both zebra finches and canaries is greater following exposure to conspecific song relative to heterospecific song exposure [6]–[8], and in zebra finches, call-induced ZENK expression is greater in the presence of conspecifics [9]. This gene induction is transient, and shows an adaptation phenomenon (originally called “habituation”) by diminishing after repeated presentations of a given song and returning when the bird hears a novel song [7].

Like the ZENK response, electrophysiological activity in zebra finch NCM is greater in response to conspecific vocalizations than to heterospecific vocalizations and other sounds [10], [11]. Furthermore, electrophysiological responses in NCM decrease following repeated presentation of a given stimulus, and vigorous responding occurs upon presentation of a novel song [12]. This decreased responding to a particular sound (a form of stimulus-specific adaptation) persists longer for conspecific song stimuli than for heterospecific song and other auditory stimuli. This pattern of specificity suggests that NCM is specialized for discrimination and memory processes that could serve species and individual recognition, based on learned vocal communication signals.

Using electrophysiological recordings in NCM, we have recently found that simple tone stimuli can be used to explore how fundamental spectral and temporal response properties underlie responses obtained with complex song stimuli [1], [12]. First, we showed that responses in NCM differ from those in the thalamo-recipient region, field L2 (on the principal input pathway to NCM). NCM sites are more broadly tuned and have more sustained responses [13]. Second, we demonstrated that temporal and spectral dynamics of auditory tuning in NCM differ between canaries and zebra finches. NCM sites in zebra finches are more broadly tuned and have more sustained responses than those in canaries [1]. Such differences could underlie the selectivity for species-typical song found in ZENK and electrophysiological studies, and may reflect species differences in vocal repertoires. Canary vocalizations include harmonically-structured notes, trills and whistles of a narrower frequency range, often with fast repetition rates, while zebra finch songs and calls are composed almost entirely of broad-band harmonically-structured notes that are seldom repeated. Broader tuning in zebra finch NCM may contribute to selectivity for broad-band zebra finch songs and calls. In contrast, narrower tuning in canary NCM could contribute to enhanced processing for the narrow-band whistles.

It is not known whether the conspecific preference found in NCM is a function of innate species-specific response characteristics of this region, or whether other proximate factors influence this selectivity. We speculated that the species differences in NCM tuning could also reflect early or even recent auditory experience, and decided to test the latter. In order to determine whether tuning differences reflect recent experience, we exposed separate groups of adult canaries and zebra finches of each sex in breeding condition to different housing environments. Birds were housed either in their home aviary with conspecifics, in an aviary with heterospecifics (canary subjects in a zebra finch aviary, and vice versa), or in isolation (Table 1) for 9 days prior to electrophysiological assessment of tuning functions using methods from our earlier studies [1], [13].

**Table 1:** Each testing group is shown, as assigned by housing condition, species, and sex.

Housing Condition	Species	Sex	Number of Subjects
CROSS	Canary	Male	10
		Female	4
	Zebra Finch	Male	5
		Female	5
NORM	Canary	Male	7
		Female	7
	Zebra Finch	Male	5
		Female	5
ISO	Canary	Male	14
		Female	5
	Zebra Finch	Male	5
		Female	5

The number of subjects in each group is shown in the 4^th^ column, and includes the number of canaries added to the original groups for the analyses of seasonal effects.

Our results were surprising in two ways; first, cross-housed birds showed clear changes in tuning that were greater in canaries than in zebra finches; and second, these changes resulted in narrower tuning in both species. Canaries, but not zebra finches, are seasonal breeders so we also tested canaries from outside of the breeding season to assess the possible effects of reproductive status on tuning widths in that species, and found that tuning width increases from the spring to later in the year only in cross-housed males.

## Methods

### Subjects

All subjects were adults, obtained either from the Rockefeller University Field Research Station, born and raised in an aviary at Rutgers University, or purchased from a local vendor. All procedures were approved by the Animal Care and Use Committee of Rutgers University. The initial experiment tested birds under breeding season conditions: the zebra finch aviary was kept under a 12:12 light-dark cycle and the canary aviary was kept under a natural light-dark cycle corresponding to the local photo-period. The species comparison experiments took place from mid-February to mid-June, a time when canaries are reproductively active in the lab. As food and water were available ad libitum, zebra finches were also housed in conditions that facilitate reproduction.

For 9 days prior to testing (7 days+2 days during recovery from pre-testing surgery), groups of birds were either left in their home aviary with conspecifics (NORM), cross-housed in an aviary with heterospecifics (canary subjects in a zebra finch aviary, and vice versa: CROSS), or placed in isolation (ISO) in custom-made soundproof boxes. Separate groups of each species and sex were tested for each of these housing conditions (12 groups altogether, (Table 1). Each group consisted of 5 animals, with the exception of the CROSS female canaries with 4 birds (data from the 5^th^ bird were omitted, as sites were histologically defined as being outside of NCM). ISO birds were kept under a light-dark cycle that corresponded with that of their home aviary. Due to differences between the aviary light cycles, CROSS zebra finches experienced a slight increase in day length (1–2.25 hrs/day) and canaries a slight decrease (1–2.25 hr/day) during their respective periods of heterospecific housing.

Canaries of both sexes were used to test the seasonal effect on tuning. For this analysis, additional birds were tested outside of the breeding season and added to the population of the initial experiment, so that the tuning results could be compared over a larger range of months. Total range: February through December, total n of males = 10 CROSS, 7 NORM, and 14 ISO; total n of females = 4 CROSS, 7 NORM, and 5 ISO.

### Apparatus and Procedure

This experiment employs methods that we developed previously for the analysis of spectral and temporal response properties to tone stimuli in the songbird auditory telencephalon [1], [12]. To prepare for recording, animals were anesthetized (Nembutal 50–55 mg/kg, Abbot Laboratories, N. Chicago, Ill), placed in a stereotaxic device, and a metal head post was attached to the skull with dental cement (Dentsply Caulk, Milford DE). The head post and a custom-made body tube permitted comfortable immobilization of awake animals during testing. 48 hrs later, birds were tested in an acoustically isolated sound booth (IAC Inc., Bronx, NY). A multielectrode microdrive (Thomas Recording, Giessen, Germany) controlled the depth of seven quartz-platinum/tungsten microelectrodes, allowing for simultaneous recording at multiple sites (3 in the left hemisphere, 4 in the right). Recorded activity was amplified (total gain: 19,000) and filtered below 500 Hz and above 5 kHz (Brownlee Model 440); All amplified activity consisted of spikes from a small number of multiple, non-isolated units near the recording tip of each electrode.

In order to define the location of recording electrodes along the caudo-rostral and medio-lateral axes, a 150 square, mesh gilded nickel grid (Electron Microscopy Sciences, Fort Washington, PA) was glued over the skull opening (inter-hole distance: 170 microns). Microelectrodes (Type ESI2ec, impedance: 2–4 M ohm, Thomas Recording) were then guided into specific grid holes. Electrode depths relative to the brain surface were independently controlled by the multielectrode microdrive. Combining grid and microdrive calibration allowed for the identification of recording locations from all sites in NCM. In addition, following each recording experiment electrolytic lesions were made by passing current (20 µA for 10 secs) through the recording electrodes at select sites. Three lesions were made in each hemisphere: one electrode made a single lesion and a second electrode made two lesions separated in depth by at least 500 microns. Animals were sacrificed by Nembutal overdose (10 ml/mg) and perfused with saline (0.9%, 40 ml) followed by paraformaldehyde (3.3%, 20–40 ml). Fixed brains were removed and cut on a vibratome into 50 µm parasagittal sections, then processed for Cresyl violet histology.

In initial penetrations, white noise stimuli with the amplitude envelope of canary song were used to search for responsive sites from each of the electrodes. Recordings were then made either at this first set of sites or at sites that were deeper by a predetermined amount (200–500 microns deeper than the first responsive site). Initial recording depth was varied across subjects in order to avoid a strong bias for dorsal sites. The tuning set was played at the initial recording sites and then repeated at successive depths (in 200–500 micron increments) along the dorso-ventral axis. After the first set of penetrations, the electrode array was repositioned and a new set of tuning penetrations was made.

### Stimuli

A set of 20 tone stimuli consisted of sine wave bursts with tapered onsets and offsets (cosine taper duration: 5 ms; frequency range: 250–5000 Hz in 250 Hz increments; duration: 250 ms; sample rate: 40 KHz) generated in SIGNAL 4.0 for Windows (Engineering Design, Belmont, MA). Stimuli were equated for RMS amplitude and their effective amplitude on playback was well above threshold (range: 50–68 dB, “A” scale). The stimulus set was presented in pseudo-random (shuffled) order through a speaker (inter stimulus interval: 6s) under computer control (Spike2 version 5.05, Cambridge Electronic Design, Cambridge, UK). All frequencies were repeated 3 times per recording site. The responses for each frequency at each site were averaged. Presentation of all stimuli occurred in a single 1–2 hour recording session.

### Data analysis

**Figure 1:**
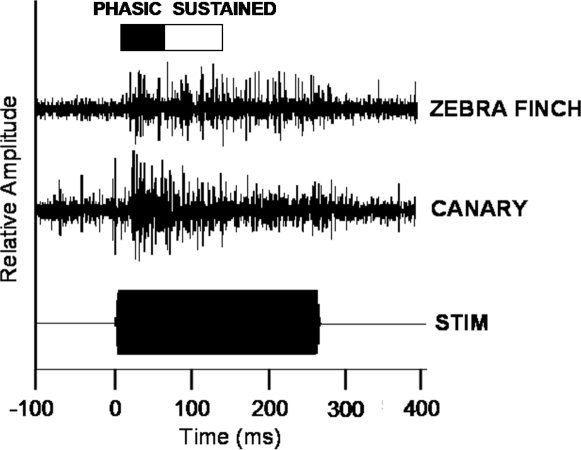
Representative multi-unit response traces from NCM in a zebra finch male (top) and a canary male (middle) in response to a tone stimulus. The stimulus is shown as an amplitude envelope trace (bottom). The phasic (black) and sustained (white) recording windows are shown at the top. Sites respond 10–20 ms after stimulus onset, and responding persists into the sustained period (60–130 ms). Zebra finch responses are usually greater in the sustained period than those of canaries (adapted from Terleph, Mello, and Vicario 2007).

A root-mean-square (RMS) value was obtained during the baseline period of each trial (a 500 ms window occurring prior to stimulus onset), and during separate response windows described below. To quantify the RMS, each digitized value is squared, the mean of these squares over the response interval is computed, and the square root of that mean is taken. This provides a method of rectifying the multi-unit activity and computing its average power. The RMS time windows used for statistical comparisons of within-stimulus tuning width occurred at 2 times, designated as phasic and sustained: 1) phasic responses were the difference between RMS over a 50 ms window occurring 10 ms after stimulus onset and baseline RMS, 2) sustained responses were the difference between RMS over a 70 ms window immediately following the phasic window and the baseline RMS (Fig. 1). The duration and onset time for each sampling window were consistent with and based upon our previous analyses of response properties throughout the region. Typical responses begin 10–20 ms after stimulus onset and reach a phasic peak within the first 50 ms. The sustained window samples the post-peak plateau region of the response and was originally chosen so as to be able to measure responses to short stimulus durations. Tuning curves were obtained from phasic and sustained response windows by averaging the 3 responses to each stimulus frequency of the tuning set at a given site and plotting its amplitude. Tuning width was defined as the frequency range of a contiguous set in which each stimulus frequency elicits a response at least 1 SD above baseline, consistent with our earlier work that compared tuning between brain areas and across species [1], [13].

Tuning width of the phasic and sustained periods were analyzed separately for each species by independent measures, factorial ANOVAs that treat sex and housing condition (CROSS, NORM, and ISO) as categorical predictors. A sustained/phasic tuning width ratio was computed and a similar factorial ANOVA compared ratios by sex and housing condition. Seasonal effects on tuning were established by comparing data collected from canaries between February to December (birds from the original data set, and new birds recorded outside of breeding conditions). The date of recording was included as a covariate in ANCOVAs that treated sex and housing condition as independent variables. HSD post hoc tests were used to further analyze ANOVA and ANCOVA results.

Multiple sites were recorded from each animal, and all recording sites were considered to be independent measures in all statistical tests. It can be argued that sites from the same animal are not truly independent measures, and we therefore may have overestimated the degrees of freedom in our tests. In order to verify that there was independence between sites measured within each animal, sites within each bird in the same group were randomly paired and the tuning widths for each member of the pair was assigned to the independent variable or the dependent variable in a linear regression that included sites from all birds in the group. We did these comparisons in select groups, chosen because each contained a large number of sites that were recorded from approximately the same time of year in all animals, controlling for possible seasonal effects. Subjects were canaries in the male CROSS and ISO conditions, and female canaries in the ISO condition. The tests showed no significant relationship between the pairs of sites across birds in each stimulus condition (p = 0.24, 0.13, 0.70 for each group, respectively). For a further comparison, all sites from these same birds were combined into a common pool of sites. Then sites were randomly paired from the entire pool and the tuning widths for each member of the pair were randomly assigned to the independent variable or the dependent variable in another linear regression. This test showed no significant relationship and a comparison of correlation coefficients from the two tests showed no difference between sites paired within each bird and sites paired across birds (p = 0.24). Therefore sites within each bird appear to be independent samples.

## Results

### Recent experience affects tuning in NCM

**Figure 2:**
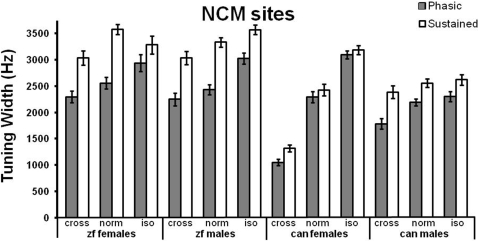
Tuning width in NCM for zebra finches and canaries of each sex, and in each housing condition (CROSS, NORM, and ISO). Tuning width is compared across phasic response windows (gray bars) and sustained response windows (white bars). Overall tuning is broader in ISO birds, and narrower in CROSS birds, relative to NORMs. Activity during the response interval that was more than 1 SD above control level was considered a response. The tuning width was estimated as the largest number of contiguous frequencies in the stimulus set that elicited such a response. That number was multiplied by the spacing of the stimuli in the set (250 Hz) to produce a measure of tuning width. As reported previously (Terleph et al., 2007), overall tuning is broader for zebra finches than canaries. However, species differences are more pronounced between the CROSS groups, and the tuning width of ISO canary females is comparable to that of zebra finches. Error bars represent ±1 SEM of all recording sites in each region.

We predicted that there would be either no effect of heterospecific auditory exposure or that any changes would cause tuning to resemble the species to which the bird had been recently exposed, i.e, that tuning in canaries would broaden to resemble zebra finch tuning and zebra finch tuning would narrow to resemble canary tuning. To our surprise, we not only saw clear differences in auditory tuning between groups exposed to different social/acoustic environments, but also found that the environment had a similar effect in both species. Figure 2 shows phasic tuning widths (gray bars) and sustained tuning widths (white bars) from male and female zebra finches (left) and canaries (right) for each housing condition (CROSS, NORM, and ISO), recorded during photoperiods associated with the breeding season. Table 2 shows all significant effects from ANOVAs that compared tuning widths within each species, and for both the phasic and sustained portions of responses. Each ANOVA compared sex and housing condition, and all ANOVAs found a difference between the housing conditions. The general trend was narrower tuning in CROSS birds and broader tuning in ISOs, relative to those housed in their home aviary (NORM). Post hoc tests show that during the phasic period CROSS birds had narrower tuning in NCM than ISOs for each sex, and in both species. Tuning during the sustained period shows a less robust effect, although the general trend is similar: CROSS<NORM<ISO tuning width.

**Table 2:** Significant results of ANOVAs for all birds tested under breeding conditions.

Dependent Variable	Significant Effect	F statistic	Probability
Phasic width (Zebra finch)	Housing condition	F(2, 525) = 14.6	p<0.0001
Sustained width (Zebra finch)	Housing condition	F(2, 525) = 7.0	p<0.0001
Sustained/Phasic ratio (Zebra finch)	Housing condition	F(2, 525) = 10.2	p<0.0001
Phasic width (Canary)	Housing condition	F(2, 649) = 73.0	p<0.0001
	Condition x Sex	F(2, 649) = 26.7	p<0.0001
Sustained width (Canary)	Housing condition	F(2, 649) = 39.0	p<0.0001
	Sex	F(1, 649) = 5.3	p<0.05
	Condition x Sex	F(2, 649) = 23.8	p<0.0001
Sustained/Phasic ratio (Canary)	Housing condition	F(2, 649) = 5.5	p<0.01
	Sex	F(2, 649) = 9.3	p<0.01

Independent measures, factorial ANOVAs treat sex and housing condition (CROSS, NORM, and ISO) as categorical predictors. A sustained/phasic tuning width ratio was computed and a similar factorial ANOVA compared ratios by sex and housing condition.

The tuning changes from the phasic to sustained periods were assessed by computing the ratio of sustained to phasic tuning width for each site, then comparing these ratios across housing conditions in each species. Each species showed a significant difference between housing conditions (Table 2). The overall trend for canaries of both sexes was higher ratios in the CROSS group and lower in the ISO, relative to NORM, indicating that tuning became relatively wider in CROSS birds from the phasic to sustained period, and showed little change in the ISOs. Group differences therefore decreased during the sustained period, as reported above.

It is possible that tuning could have been wider in ISO birds because of increased sensitivity to sounds in general, regardless of frequency, producing larger responses at marginal frequencies and increasing the measured tuning width. In order to evaluate whether or not this was the case, the absolute amplitude of responses to the preferred frequency at each site (the frequency that evoked the highest response amplitude) was compared between the groups that differed the most in their tuning: the CROSS and ISO canary females tested under breeding conditions. All amplitudes were normalized by computing the ratio of the response amplitude (minus its pre-stimulus baseline) to the pre-stimulus baseline amplitude [(post stimulus amplitude−baseline)/baseline]. Independent samples t-tests show that the amplitudes of responses to preferred frequencies do not significantly differ (CROSS vs. ISO phasic amplitudes: T = 0.32, p = 0.75; CROSS vs. ISO sustained amplitudes: T = 0.63, p = 0.53). Thus, increased sensitivity to all frequencies is not observed and cannot explain the increased tuning width in ISO birds.

### The effect of recent experience on tuning differs between sexes

The largest sex difference in tuning width occurs in canaries: during the phasic period, there is a housing condition x sex interaction, and during the sustained period there is a significant sex effect and a housing condition x sex interaction (Table 2). In each case, these differences reflect a stronger housing condition effect (CROSS<NORM<ISO) in females than males. Tuning during the phasic period differed between each group of female canaries (CROSS<NORM<ISO, p<0.0001 in each case). For male canaries, phasic tuning was narrower in CROSS birds relative to NORMs (p<0.05), and ISOs (p<0.01), and ISOs did not significantly differ from NORMs (Figure 2). Phasic tuning in zebra finch males was wider for ISO than NORM (p<0.01) and CROSS birds (p<0.01), but CROSS males did not differ from NORMs. Phasic tuning was narrower in CROSS zebra finch females than ISOs (p<0.05), but neither differed from NORMs (Figure 2).

For comparisons of the sustained period, post hoc tests reveal that Canary females differ across all housing conditions (CROSS<NORM<ISO, p<0.0001 in each case). Male canaries, however, do not differ across groups during the sustained period (Figure 2). Zebra finches of either sex show little difference in sustained tuning width between housing conditions, although CROSS females do have narrower tuning than NORMs (p<0.05).

Canaries also showed a sex difference in the Sustained/phasic tuning width ratio, with a higher ratio in males: Tuning widened from the phasic to sustained period in males relatively more than in females. The width ratio was highest for CROSS male canaries, and significantly differed from that of ISO and NORM females (P<0.0001 and 0.01, respectively), but from no other groups. Zebra finches also showed the lowest Sustained/phasic ratio in the ISO group, but their highest ratio was in the NORM group (highest for females). However, post hoc tests reveal no group differences within each sex for canaries or zebra finches, with the exception of a higher ratio for NORM female zebra finches relative to ISOs (P<0.05).

### Seasonal Changes in Plasticity

The analyses so far have presented data from the main experiment, in which canaries were recorded during the breeding season. Because canaries are seasonal breeders, and because seasonal changes in brain anatomy have been reported in this species [14], [15], we expanded our sample to include canaries that were exposed to altered auditory experience outside of the breeding season. ANCOVAs performed on this larger sample of male and female canaries found that the housing condition, date and the interaction of these two factors have significant effects on phasic and sustained tuning widths of canary NCM. Tuning width increases from the spring to later in the year, but this overall seasonal effect is significant only in the CROSS, and not in the NORM or ISO conditions. As with the smaller sample of birds from the breeding season reported above, ANCOVAs analyzing both phasic and sustained response data showed significant main effects for condition (Phasic: F = 20.0, p<0.001, Sustained: F = 9.1, p<0.001). Again, the general trend in each case is narrower tuning in CROSS groups and broader tuning in ISO groups, compared to NORMs. Post hoc tests for phasic responses reveal that tuning is wider for the ISO group than the CROSS group for males (p<0.01), but the NORM group is not significantly different the others. In contrast, all three groups of females significantly differ (p<0.0001 in each case), although there is no significant main effect for sex or sex by housing condition interaction. Post hoc tests of the sustained responses showed no difference across groups in males but significant differences between each group of females (p<0.0001 in each case). The trend observed in the smaller group of breeding season birds holds when birds from outside of the breeding season have been added to the sample: the housing condition has a greater effect on females than males and a greater effect on phasic tuning width than sustained.

**Figure 3:**
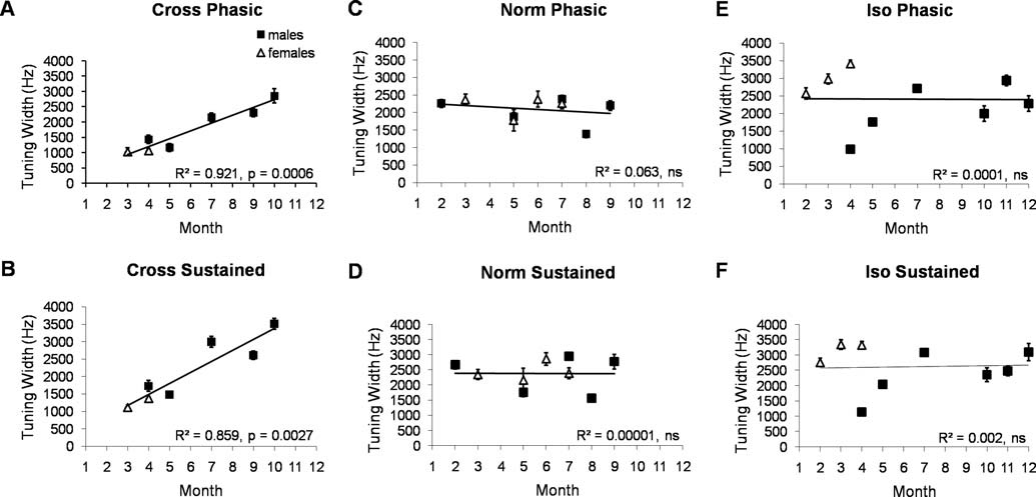
Tuning width of Phasic (Top) and Sustained (Bottom) responses from the CROSS (A and B), NORM (C and D) and ISO (E and F) canary groups, respectively, by month of the year. Black squares represent the average tuning width of all sites recorded from males in a given month and open triangles represent the average tuning width of all sites recorded from females in a given month, and error bars represent ±1 SEM. The average tuning width of NORM canaries in the month of March is omitted, as only 3 sites from a single bird were recorded during that month. Regressions are drawn for each figure (data from both sexes included). R_2_ values and probabilities are indicated for each scatter plot, and correspond with the regressions drawn to the average values for each month, and in both sexes. Canaries show a significant effect of season on tuning in the CROSS condition only, during both the phasic and sustained periods. Sex differences, such as those in the ISO group, are discussed in the text.

ANCOVA analyses of both phasic and sustained responses showed a significant main effect for date (Phasic: F = 40.6, p<0.001, Sustained: F = 39.9, p<0.001). Separate linear regressions that treat either the phasic response or the sustained response as a dependent variable showed that tuning width increased from the spring to later in the year (Phasic: R^2^ = 0.005, p<0.05, Sustained: R^2^ = 0.016, p<0.001). Both ANCOVAs also showed an interaction between date and housing condition (Phasic: F = 23.6, p<0.001, Sustained: F = 8.07, p<0.001) The linear regressions for each housing condition showed that the seasonal effect is only significant in the CROSS group (Phasic: R^2^ = 0.275, p<0.0001, Sustained: R^2^ = 0.302, p<0.0001, with CROSS birds showing a robust effect of housing condition as a function of the time of year, while the NORM and ISO birds did not. Figure 3 shows these data averaged across all females and males for each month of testing. Regressions drawn to these averaged data show even greater R^2^ values, in the CROSS housing condition only. Therefore, the overall seasonal effect can mainly be explained by the seasonal effect in the CROSS group.

### Seasonal plasticity and sex differences

The ANCOVA that analyzed phasic responses showed a significant sex by date interaction (F = 18.42, p<0.001), consistent with linear regression data that showed the phasic response significantly varied by date for males (R^2^ = 0.037, p<0.0001), but not females. There was no sex by date interaction in the sustained response. A linear regression also showed a date effect in the sustained responses of males (R^2^ = 0.036, p<0.0001), but not females.

ANCOVAs also showed a significant interaction of sex by date by housing condition (Phasic: F = 36.6, p<0.001, Sustained: F = 22.6, p<0.001). Linear regressions analyzing the phasic responses showed a significant date effect in the male CROSS and the male ISO groups (R^2^ = 0.16, p<0.0001; and: R^2^ = 0.073, p<0.0001, respectively). In contrast, in females the date effect is only significant in the ISO group (R^2^ = 0.136, p<0.001). The linear regressions of sustained responses showed a significant date effect for the male CROSS group and the female ISO group (R^2^ = 0.160, p<0.0001; and R^2^ = 0.042, p<0.05, respectively).

The seasonal effect is most pronounced in the male CROSS group. However, a lack of seasonal effect in the female CROSS group is likely due to the fact that it was a smaller sample, and these females were only recorded from March 24–April 28. In fact, a comparison of only the CROSS males and females that were recorded in the spring reveals only a weak sex difference, and only in the sustained response (F = 5.3, p<0.05, Fig. 2). Tests of more CROSS females outside of the breeding season might reveal less of a sex difference. No sex difference in tuning width exists in the NORM condition (Fig. 3C and D). However, a large difference exists between ISO males and females recorded during the spring (F = 146.7, p<0.0001, Figs. 2 and 3E and F). This sex difference in the ISO group during the spring contributes most to the sex by date by condition interaction.

## Discussion

These findings demonstrate that NCM properties are not fixed in the adult bird: auditory tuning widths are influenced either by recent acoustic experience, social influences, or both, in canaries and zebra finches of both sexes. This short-term neural plasticity effect is largest during the phasic portion of responses. Exposure to a novel environment increases spectral selectivity in NCM relative to a familiar conspecific environment, and isolation may decrease it. The plasticity differs between species and sex, with the largest apparent sex difference in canaries, where the effect was most pronounced in females. However, a strong seasonal influence was found on the tuning width plasticity in CROSS male canaries.

Interestingly, and counter to our initial predictions, the direction of change in tuning width was not dependent upon the different acoustic environments of CROSS canaries and zebra finches. We had predicted that canaries housed in the zebra finch aviary would show broader tuning (more like that of a zebra finch), and cross-housed zebra finches would show the reverse. Instead, CROSS birds of both species showed narrower tuning, which seems to depend upon the novelty of the housing environment rather than its spectral features. In contrast, the ISO condition resulted in wider tuning, suggesting that NORM tuning is actually maintained by input from other birds, and “relaxes” in isolation. The tuning changes seen in both CROSS and ISO birds suggest that songbirds adjust spectral tuning rapidly when adapting to novel acoustic and/or social environments. Sharper tuning may aid in the detection and discrimination of specific spectral bands in novel stimuli, while larger effects in the phasic period may allow finer temporal resolution of spectral transitions, especially at sound onsets.

Factors not directly related to the specific spectro-temporal aspects of auditory input may have led to the observed changes in NCM tuning. A change in social environment potentially results in a broad range of influences. The immediate social environment has been shown to influence NCM activity, as zebra finches show an increase in the ZENK response to calls when in the presence of conspecifics [9]. However, the animals we studied, although subject to recent social manipulation, were tested in isolation. Therefore the changes observed did not rely upon the immediate presence of other animals. Manipulations such as cross housing and isolation may differ in their respective roles as potential stressors, inducing changes in hormonal condition. This could account for the larger effect of social manipulation upon the tuning width in canaries. Unlike zebra finches, that are tolerant of the presence of others and are often found in large flocks [16], canaries are less gregarious. Although they do not possess large territories, socially monogamous wild canaries do defend their nest during the breeding season [17], [18]. Due to such behavioral differences, canaries might normally attend closely to the songs and calls from a smaller number of neighbors than the zebra finch does. Radical changes to a canary's auditory and/or social environment might therefore have a larger influence. Despite the fact that both the ISO and CROSS conditions might be considered socially novel and potentially stressful, the tuning changes produced by the ISO condition (broadening) are opposite in direction to the changes in the CROSS condition (narrowing) for each species. Thus, no simple theory of novelty or stress can account for the results. In order to tease apart the relative contributions of social and auditory influences on NCM tuning, it will be necessary in future studies to selectively manipulate the social and acoustic environments of subjects in a more controlled and independent way.

We have found that both the plasticity of NCM tuning and the sex difference in that plasticity were less pronounced in zebra finches. The smaller degree of plasticity in the tuning of zebra finch NCM could be because songs become fixed in this species: in contrast to canaries, male zebra finches learn their adult song only once (during the 1^st^ year of life), and their song is not as complex as that of canaries. We only tested adults in this study, so we have not explored the interesting possibility that experience-dependent plasticity of NCM tuning might be similar in juvenile zebra finches and canaries, as juveniles of both species are still learning song.

Narrowed tuning in NCM could be a marker of improved learning and/or discrimination of new songs, but it would be necessary to perform psychophysical/behavioral studies to demonstrate such a relationship. The species differences that we have reported here and in previous work [1] are correlations that only suggest such a relationship. If NCM tuning does reflect such discrimination, then the fact that a sex difference was absent in canaries only in the NORM group might be explained by the fact that there would be no need for new song discrimination learning in this group, as their environment was unchanged. However, a significant sex difference was found between ISO males and females recorded under breeding conditions, suggesting that female canaries may be more sensitive to isolation than male canaries and zebra finches of either sex, or that the hormonal state of males during the spring overrides an isolation stress effect that only occurs in females at this time of year. Alternatively, males recorded in the spring from the ISO group might have been singing (females typically do not sing), and this auditory input could have maintained NCM tuning sufficiently to mask the isolation effect.

A seasonal influence on tuning width plasticity was observed in canaries tested across breeding and non-breeding conditions and this effect was strongest in the CROSS males. Tuning width was narrower early in the year (between February and May) than later. This late winter-to-spring period is early breeding season for canaries, when males sing the most, and add new song syllables to their individual song repertoires. Testosterone levels also increase during this period [19]. This seasonal effect suggests that auditory plasticity in NCM may be modulated by a combination of sex hormones and novel auditory and/or social stimulation. The seasonal effect was not observed in NORM canaries, suggesting that testosterone by itself may be necessary, but not sufficient to induce changes to NCM spectral selectivity.

Although the seasonal effect was not observed in female CROSS or ISO canaries, we cannot rule out the possibility that this effect also exists in females, but was undetected, due to the small sample size and narrow seasonal range sampled in CROSS females. The tuning in female canaries was, however, strongly influenced by recent experience. This might be due to female canaries closely attending to the vocalizations of male suitors during the breeding season, to help in selecting an appropriate mate. The sex differences we report are part of a growing body of findings that suggest a role for sex steroid hormones in local auditory processing in NCM. For example, in female white-throated sparrows (*Zonotrichia albicollis*), induction of the activity-dependent *zenk* gene is selective for song only when plasma estradiol exceeds non-breeding levels [20]. It is not known if the species and sex differences observed are due to the underlying physiology or functional organization of NCM, or if they originate elsewhere in the ascending auditory pathway. However, known sex differences do exist in a specific subset of GABAergic neurons in zebra finch NCM [21], and the distribution of these neurons is similar to that of mRNA expression for the estrogen-generating enzyme aromatase [22]. GABAergic neurons make up about half of all neurons in NCM [22], [23], and many of these express the activity-dependent gene zenk following song-stimulation. Such neurons could potentially play a role in NCM response plasticity; studies in other species suggest that experience-dependent modification in the tuning of auditory neurons involves changes in GABAergic transmission [24]–[27].

In summary, the tuning properties of NCM are not stable, but show rapid and robust plasticity. We do not yet know how quickly these changes occur, and thus cannot speculate about the cellular mechanism, but a rapid influence of recent sensory experience on context-specific receptive fields has been described in the visual cortex (review: [28]). The species difference in tuning we observed earlier [1] does not document a fixed property in the adult bird and the neural selectivity is not tuned to match the acoustic features of conspecific vocal repertoires in a simple way. Although zebra finches became more canary-like (narrower tuning in NCM) when housed with canaries, canaries did not become more zebra finch-like, so the contribution of recent experience to species differences in tuning (our original question) is hard to quantify. Nonetheless, this lability is likely to influence the results of electrophysiology and immediate early gene studies that do not control for recent experience, including paradigms that have tested conspecific selectivity using natural vocalizations as stimuli [6], [9], [11], [29]. These findings suggest the importance of documenting recent experience and implementing controls for housing conditions (e.g. when isolation is part of the protocol), auditory exposure and season of testing when characterizing NCM responses to external stimuli. A significant impact of recent experience may also apply to other levels of organization, from brain and behavior to gene expression, but is often neglected in experimental protocols.

At the same time, the plasticity of auditory responses in NCM provides an experimental variable that can be used to probe the mechanisms that relate neuronal processing to behavior. For example, our findings suggest a role for seasonal modulation of auditory processing that may reflect behavioral state and influence reproductive choices. Rapid neural plasticity may both reflect and influence the discrimination and memorization of complex song stimuli, especially when birds encounter a new social and auditory environment at the start of the breeding season. We know that NCM neurons show song-specific adaptation that can be thought of as a recognition memory for the songs of other individuals. Exposure to the songs of a social group when the bird is in the appropriate hormonal condition may result in a system of memories that generalizes and categorizes these songs to map the current auditory landscape in the service of adaptive behavior, e.g. mate choice or song imitation. More generally, at any given moment, NCM auditory selectivity reflects the modulatory effect of experience on a set of initial conditions (perhaps innate) and can be thought of as a set of dynamic perceptual filters through which the animal hears the world.
